# Characterization of Brain–Heart Interactions in a Rodent Model of Sepsis

**DOI:** 10.1007/s12035-016-9941-z

**Published:** 2016-05-26

**Authors:** Bernardo Bollen Pinto, Cristiane Ritter, Monique Michels, Nicolò Gambarotta, Manuela Ferrario, Felipe Dal-Pizzol, Mervyn Singer

**Affiliations:** 1 Bloomsbury Institute of Intensive Care Medicine, Division of Medicine, University College London, Cruciform Building, Gower Street, London, WC1E 6BT UK; 2 Department of Anaesthesia, Emergency, and Intensive Care, Centro Hospitalar do Porto, Largo Prof. Abel Salazar, 4099-001 Porto, Portugal; 3 Department of Anaesthesia, Pharmacology and Intensive Care, Geneva University Hospitals, Rue Gabrielle-Perret-Gentil 4, 1205 Geneva, Switzerland; 4 Laboratory of Experimental Pathophysiology, Graduate Program in Health Sciences, University of Southern Santa Catarina, Av Universitária, 1105, Criciúma, 88806000 SC Brazil; 5 Department of Electronics, Information, and Bioengineering (DEIB), Politecnico di Milano, Via Ponzio 34/5, 20133 Milan, Italy

**Keywords:** Sepsis, Inflammation, Encephalopathy, Autonomic nervous system, Heart rate variability, Contractility

## Abstract

Loss of heart rate variability (HRV) and autonomic dysfunction are associated with poor outcomes in critically ill patients. Neuronal networks comprising brainstem and hypothalamus are involved in the “flight-or-fight” response via control over the autonomic nervous system and circulation. We hypothesized that sepsis-induced inflammation in brain regions responsible for autonomic control is associated with sympathovagal imbalance and depressed contractility. Sepsis was induced by fecal slurry injection in fluid-resuscitated rats. Sham-operated animals served as controls. Echocardiography-derived peak velocity (PV) was used to separate septic animals into good (PV ≥0.93 m/s, low 72-h mortality) and bad (PV <0.93, high 72-h mortality) prognosis. Cytokine protein levels were assessed by ELISA. All experiments were performed at 24 h post-insult. Increased levels of inflammation and oxidative injury were observed in the hypothalamus (TNF-α, IL-10, nitrite and nitrate and carbonyl groups) and brainstem (IL-1, IL-6, IL-10, nitrite and nitrate and carbonyl groups) of the septic animals (*p* < 0.05 vs. sham), but not in the pre-frontal cortex, an area not directly implicated in control of the autonomic nervous system. Good prognosis septic animals had increased sympathetic output and increased left ventricular contractility (*p* < 0.05 vs. sham). There was a significant inverse correlation between high frequency power (a marker of parasympathetic outflow) and contractility (*r* = −0.73, *p* < 0.05). We found no correlation between the degree of inflammation or injury to autonomic centers and cardiovascular function. In conclusion, control of autonomic centers and cardiac function in our long-term rodent model of sepsis was related to clinical severity but not directly to the degree of inflammation.

**Electronic supplementary material:**

The online version of this article (doi:10.1007/s12035-016-9941-z) contains supplementary material, which is available to authorized users.

## Introduction

The central nervous system (CNS) controls a range of physiological functions crucial for the body to maintain a coordinated response to injury and stress [1]. Cerebral networks passing through the brainstem (e.g., locus coeruleus, rostral ventrolateral medulla, medullary autonomic nuclei, or parabrachial nuclei) and hypothalamus (paraventricular or supraoptic nuclei) are involved in control of sympathetic and parasympathetic output to the cardiovascular system and adrenal gland and are particularly important in establishing an adequate hemodynamic response (the “fight-or-flight” response).

However, as with any other organ, the brain itself is not spared from injury and systemic inflammation. Sepsis-associated encephalopathy is a complex disorder with a prevalence of 9–71 % [2, 3], the severity of which has been correlated with poor outcomes [2, 3]. Excess cytokines [4, 5], prostaglandins [6], reactive nitrogen, and oxygen species [7, 8] are associated with brain injury in various models of acute inflammation and sepsis. In a postmortem study, cardiovascular autonomic centers from septic shock patients manifested greater degrees of ischemia and neuronal and glial apoptosis compared to control, non-septic patients [9]. Although our understanding of the consequences of this injury pattern is far from complete, modulation of neurotransmission [10] and hormone release [11] occurs as a direct consequence of brain inflammation.

Cardiovascular autonomic failure, a poorly characterized syndrome of refractory hypotension and abrupt variations in heart rate, is associated with decreased survival in critically ill patients [12, 13]. Loss of heart rate variability, a measure of autonomic output to the heart, is observed in critically ill patients with multiple organ dysfunction [14] and sepsis [13]. Measures of sympathovagal imbalance (e.g., reduced low frequency/high frequency ratio) are strong predictors of ICU mortality [13]. Whether this association is purely epiphenomenal or contributes to poor outcomes remains to be elucidated. Mechanisms by which autonomic dysfunction possibly lead to increased ICU mortality remain largely unknown, as does the hierarchical relationship between injury in the septic brain, loss of heart rate variability, and hemodynamic failure.

Using an established fluid-resuscitated rat model of fecal peritonitis, we conducted a series of experiments to determine the relationship between injury to cardiovascular autonomic centers, heart rate variability, and septic cardiomyopathy. We hypothesized that, in sepsis, increased inflammation in brain centers responsible for autonomic control is associated with sympathovagal imbalance and depressed cardiac contractility and that these centers are affected more in animals with severe disease.

## Materials and Methods

All animal procedures were conducted in accordance with UK Home Office guidelines under the 1986 Scientific Procedures Act and following approval from the University College London Ethics Committee. Animals were maintained under artificial day–night cycles (12-h light–dark cycles; 23 ± 1 °C room temperature, 30–60 % environment humidity), received a standard diet and water ad libitum, and were allowed to adapt to laboratory conditions for at least 6 days prior to experimentation. All interventional procedures were carried out under aseptic conditions with body temperature maintained at 37 °C.

### Rat Model of Fecal Peritonitis

This model has been extensively described elsewhere [15]. In brief, under a short period of light inhalational anesthesia (2 % isoflurane), male Wistar rats 12–14 weeks old and with approximately 300 g body weight underwent right jugular vein and left carotid artery cannulation using 0.96-mm external diameter tubing (Biocorp Ltd, Huntingdale, Australia). These catheters were sited through a small neck incision, tunneled subcutaneously to emerge at the nape of the neck, and subsequently mounted onto a swivel-tether system (Instech, Plymouth Meeting, PA, USA). This allowed the rat, on recovery from anesthesia, to have unimpeded movement in its cage, free access to food and water, and intravenous fluid administration. The arterial line was connected to a pressure transducer for continuous monitoring of mean arterial pressure recorded using a 16-channel Powerlab system and Chart 7.0 acquisition software (Powerlab, AD Instruments, Chalgrove, Oxon, UK). A subcutaneous buprenorphine injection (0.05 mg/kg) (Vetergesic, Reckitt Benckiser, York, UK) was administered for pain relief prior to cessation of anesthesia. Catheters were kept patent with 0.1-ml/h heparinized saline.

After overnight recovery, sepsis was induced by intraperitoneal (i.p.) injection of fecal slurry (3 ml/kg body weight) administered through a 19G needle into the right lower quadrant of the abdomen, with care taken to not perforate the adjacent bowel. Standardized human slurry was kindly donated by the Department of Anesthesiology and Intensive Care at Friedrich Schiller University Hospital, Jena, Germany [16]. Sham animals (control) received an equivalent volume of 0.9 % saline. Two hours post-injection of slurry/saline, a continuous infusion of glucose and 6 % hydroxyethyl starch 130/0.4 (Volulyte, Fresenius Kabi, Bad Homburg, Germany) (1:1) was started at 10 ml/kg/h through the internal jugular line. A clinical scoring system [17] was used to assess overall disease severity at regular intervals. Animals were marked for absence (0), presence (1) or marked presence (2) of hunched position, bloated abdomen, conjunctival injection, piloerection, lack of movement, and lack of alertness.

### Echocardiography Measurements and Prognostication of 3-Day Outcome

Echocardiography was performed at 24 h after peritoneal injection of fecal slurry or n-saline in spontaneously breathing rats under light (1.5 %) isoflurane anesthesia using a 14-MHz probe connected to a Vivid 7 Dimension machine (GE Healthcare, Chalfont St. Giles, Bucks, UK). Pulsed wave Doppler was used to measure aortic blood flow velocity in the proximal ascending aorta, immediately before the bifurcation of the right carotid artery. Stroke volume was calculated as the product of the velocity time integral (VTI) averaged over one respiratory cycle and aortic cross-sectional area. In rats of similar age, aortic diameter was measured at 2.6 mm [18]. The average peak-to-peak distance and maximum velocity over one respiratory cycle were used to measure heart rate and peak (systolic blood flow) velocity (PV), a marker of left ventricular contractility [19]. Cardiac output (CO) was calculated as the product of stroke volume and heart rate. Previous work from our lab using the same peritonitis model during 3-day follow-up demonstrated that an aortic blood flow peak velocity cutoff of 0.93 m/s measured at 24 h was a good discriminator of 72-h survival (AUC 0.84 ± 1, *p* = 0.03) [20] and Bollen Pinto et al. (submitted). Thus, a non-invasive measure of cardiac function performed at 24 h could be used to grade septic animals according to disease severity and prognosis, thus helping to investigate mechanisms potentially involved in survival to a septic insult.

### Electrocardiography and Heart Rate Variability Analysis

Twenty-four hours post-insult, electrocardiographic (ECG) recordings were performed in spontaneously breathing rats under light (1.5 %) isoflurane anesthesia using a 16-bit PowerLab system. Recordings were made over a 6–8-min duration. Customized needle electrodes were inserted subcutaneously in the limbs, and standard lead II ECGs were recorded with LabChart software 7.0 using a sampling rate of 1 KHz. All ECGs were stored and analyzed off-line using Matlab R2014 (The MathWorks, Natick, MA, USA). R peaks were automatically detected, and the obtained RR series were then de-trended and resampled at 10 Hz to obtain evenly spaced time intervals. For each observational period, 3-min 50 % overlapping segments were analyzed. For each segment, a spectral analysis was performed by using an autoregressive model, with the following indices quantified: power in the very low frequency band (VLF, 0 Hz < f ≤ 0.2 Hz), in the low frequency band (LF, 0.2 Hz < f ≤ 0.75 Hz), and in the high frequency band (HF, 0.75 Hz < f ≤ 3 Hz); the LF/HF ratio; LF power in normalized units (LF%) computed as LF / (total power − VLF) × 100; HF power in normalized units (HF%) computed as HF / (total power − VLF) × 100; and total power [21]. For each observational period, the indices estimated in 3-min segments were averaged and considered for successive analyses.

### Serum Hormone Assays

At 24 h post-insult, a thoracotomy was performed under isoflurane anesthesia upon loss of the pain reflex. Blood samples were obtained from the chest cavity after removal of the heart and lungs (used for other studies). This approach was chosen given our observation in previous studies with this model that arterial catheters in some animals had clotted at this timepoint, thus preventing blood sampling. The blood was placed into a tube containing lithium heparin and centrifuged for 10 min at 12,000 rpm. Plasma samples were frozen under liquid nitrogen and kept at −80 °C until batched measurements of norepinephrine (enzyme-linked immunosorbent assay (ELISA), Labor Diagnostika Nord, Nordhorn, Germany); vasopressin (ELISA, Assay Designs, Ann Arbor, MI, USA); corticosterone (colorimetric immunoassay, R&D Systems, Minneapolis, MN, USA); tumor necrosis factor-α (TNF-α); interferon-γ (IFN-γ); and interleukin (IL)-1β, IL-6, IL-10, and IL-17 (all ELISA, PrepoTech, Ribeirão Preto, SP, Brazil) were performed according to manufacturers’ instructions.

### Central Nervous System Inflammation and Oxidative Injury

Twenty-four hours after peritoneal injection of fecal slurry/saline, rats were anesthetized with isoflurane and a craniotomy performed immediately upon removal of the heart. Quick separation of the hypothalamus and brainstem was achieved by careful dissection. A sample from the pre-frontal cortex was obtained as a control area not directly involved in autonomic nervous system output. Samples were frozen under liquid nitrogen and kept at −80 °C until further analysis.

The total concentration of nitrite and nitrate was determined by a modified Griess reaction method and was expressed as nanomoles per milligram of protein [22]. As all nitrate had already been reduced to nitrite by the use of nitrate reductase, this represented the combined concentration of nitrite and nitrate. Determination of carbonyl groups as a marker of oxidative stress was performed based on a reaction with dinitrophenylhydrazine (Sigma-Aldrich, Saint Louis, USA), as previously described [23]. Results are expressed as nanomoles of protein carbonyls per milligram of protein. TNF-α, IFN-γ, IL-1β, IL-6, IL-10, IL-17, and brain-derived neurotrophic factor (BDNF) were measured by ELISA according to the manufacturer’s instructions (DuoSet, R&D Systems, Minneapolis, MN, USA). All samples were assayed in duplicate.

### Data Analysis and Statistical Procedures

All variables were tested for normality of distribution using the Kolmogorov–Smirnov test. For comparison of normally distributed continuous variables, Student’s *t* test was used to compare differences between sham and septic animals (disease effect) and ANOVA with post hoc Bonferroni corrections for comparisons between sham, sepsis good prognosis, and sepsis bad prognosis groups (severity effect). Non-parametric data were compared using the appropriate equivalent. Correlations tested with Spearman’s rho were used for studies of association between brain inflammation, heart rate variability (HRV), and cardiac contractility. All analyses were performed using IBM SPSS Statistics, Version 22.0 (IBM Corp, Armonk, NY, USA) and graphs built using Prism Version 6.0 (GraphPad Software, La Jolla, CA, USA). Results were expressed as mean ± SEM. Differences were considered statistically significant at *p* values <0.05.

## Results

### Model Characterization and In Vivo Myocardial Dysfunction

**Fig. 1:**
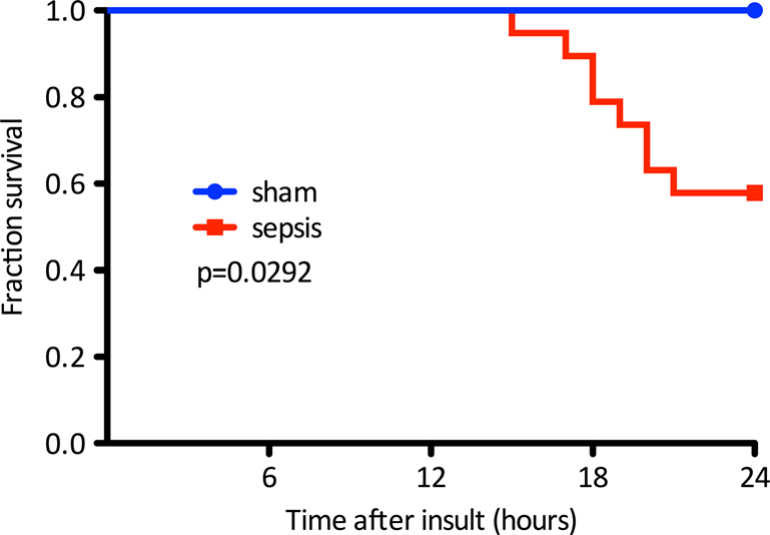
Kaplan–Meier survival curve. Legend: *p* value for log rank test (*n* = 8 for sham and *n* = 19 for sepsis)

Twenty-seven instrumented animals randomized to receive an injection of fecal slurry (sepsis, *n* = 19) or saline (control, *n* = 8) were followed for 24 h. All sham animals survived while eight (42 %) of the septic animals died before reaching this time point (Fig. 1). Clinical severity, blood pressure, and echocardiographic data recorded at 24 h are described in Table 1. Septic animals adopted a hunched position, with piloerection, a bloated abdomen, and decreased interest in their surroundings, thus presenting higher severity scores. Six (54 %) of the septic animals alive at 24 h had an aortic peak flow velocity above 0.93 m/s and were thus classified as “good prognosis,” with the remaining five being classified as “bad prognosis.”

**Table 1:** Clinical severity and hemodynamic measures at 24 h post-insult

	Sham (*n* = 8)	Sepsis	
		Good prognosis (*n* = 6)	Bad prognosis (*n* = 5)
Clinical severity (arbitrary units)	2 ± 1	3 ± 2^a^	4 ± 1^a^
Mean BP (mmHg)	121 ± 6	109 ± 5	106 ± 8
Peak blood flow velocity (m/s)	0.97 ± 0.03	1.18 ± 0.06^a^	0.85 ± 0.04^a,b^
Heart rate (bpm)	426 ± 13	451 ± 18	474 ± 14
Stroke volume (ml)	0.24 ± 0.01	0.26 ± 0.02	0.18 ± 0.01^a,b^
Cardiac output (ml/min)	103 ± 2	115 ± 5	85 ± 5^a,b^

For clinical severity score determination, animals were marked for absence (0), presence (1) or marked presence (2) of hunched position, bloated abdomen, conjunctival injection, piloerection, lack of movement, and lack of alertness (the overall score in a sum of the scores obtained for each of the six evaluated variables with highest scores corresponding to sicker animals)

*bpm* beats per minute, *m/s* meters per second, *ns* non-significant

^a^ *p* < 0.05 vs. sham

^b^ *p* < 0.05 vs. sepsis good prognosis

### Central Nervous System Inflammation and Injury

**Fig. 2:**
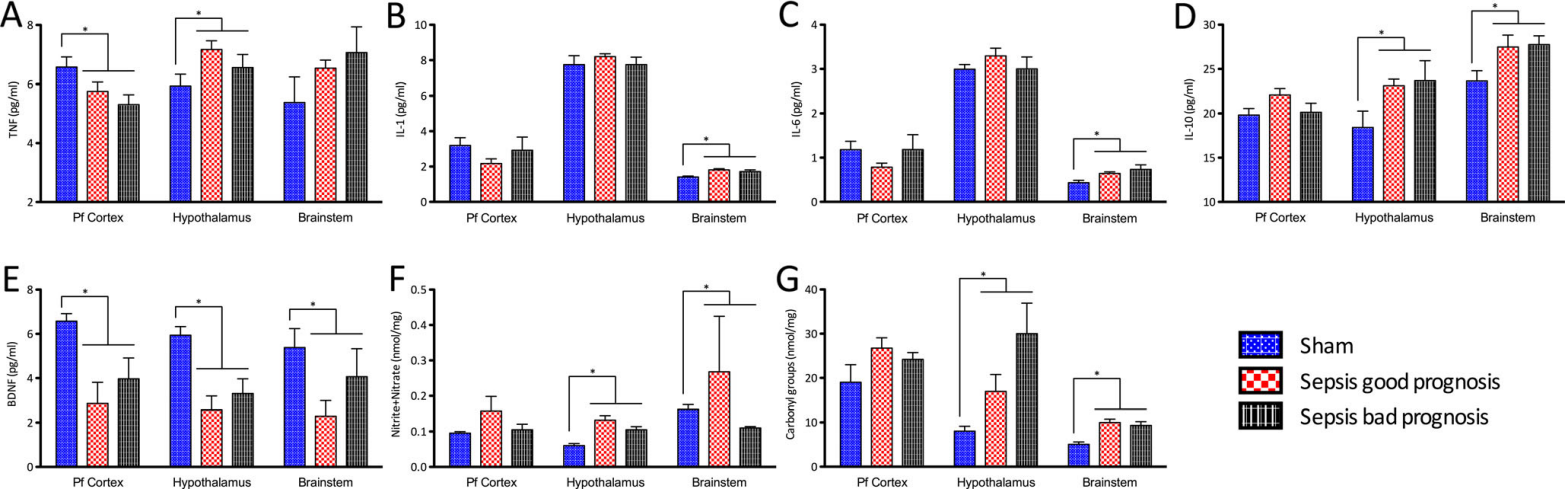
Markers of central nervous system inflammation, and oxidative and nitrosative stress. TNF-α (**a**), IL-1β (**b**), IL-6 (**c**), IL-10 (**d**), brain-derived neurotropic factor (BDNF) (**e**), nitrite and nitrate (**f**), and carbonyl groups (**g**) were measured in samples of hypothalamus and brainstem, two areas directly involved in central autonomic control (*right* of the *dashed line*) and the pre-frontal cortex (control, *left* of the *line*) in both septic and sham animals. Legend: *p < 0.05 (unpaired *t* test) (*n* = 6 for sham, *n* = 5 for sepsis good prognosis, and *n* = 4 sepsis bad prognosis)

Sepsis was associated with increased expression of inflammation markers in areas of the brain directly involved in autonomic control (Fig. 2a–d). Sepsis was associated with increased TNF-α and IL-10 levels in the hypothalamus and higher IL-1β, IL-6, and IL-10 in the brainstem. By contrast, in the pre-frontal cortex, an area not directly involved in autonomic regulation, there was no evidence of sepsis-associated inflammation. There were no differences between groups in levels of IL-17 or IFN-γ on the three brain areas studied (data not shown). Levels of nitrite and nitrate and carbonyl groups in the hypothalamus and brainstem were higher in the septic animals, suggestive of increased nitrosative and oxidative stress (Fig. 2f, g). BDNF, a mediator of neuronal plasticity, was ubiquitously depressed in the septic animals (Fig. 2e). However, contrary to our hypothesis, there was no significant severity-dependent change in any of the analyzed markers.

### Circulating Hormones, Cytokines, and Markers of Oxidative and Nitrosative Damage

In our model, there was no difference between groups in circulating adrenaline or cortisol levels at 24 h (Table 2). We found that vasopressin levels were lower in bad prognosis septic animals compared to sham control and good prognosis septic rats (*p* < 0.01).

**Table 2:** Circulating levels of stress hormones, cytokines, and markers of oxidative and nitrosative stress at 24 h post-insult

	Sham (*n* = 6)	Sepsis	
		Good prognosis (*n* = 5)	Bad prognosis (*n* = 4)
Adrenaline (pg/ml)	3.6 ± 0.6	3.9 ± 0.5	3.3 ± 0.4
Vasopressin (pg/ml)	73 ± 11	64 ± 13	22 ± 8^a,b^
Cortisol (μg/ml)	4.5 ± 0.4	3.4 ± 0.5	3.4 ± 0.5
TNF (pg/ml)	355 ± 48	265 ± 60	350 ± 55
IL-1 (pg/ml)	17 ± 4	47 ± 11^a^	13 ± 3^b^
IL-6 (pg/ml)	70 ± 28	83 ± 22	56 ± 9
IL-10 (pg/ml)	49 ± 6	105 ± 37	78 ± 27
Nitrite + nitrate (nmol/mg)	37 ± 7	98 ± 23^a^	25 ± 6^b^
Carbonyl groups (nmol/mg)	1.2 ± 0.1	2.2 ± 0.5	2.6 ± 0.7

^a^ *p* < 0.05 vs. sham

^b^ *p* < 0.05 vs. sepsis good prognosis

### Heart Rate Variability

**Fig. 3:**
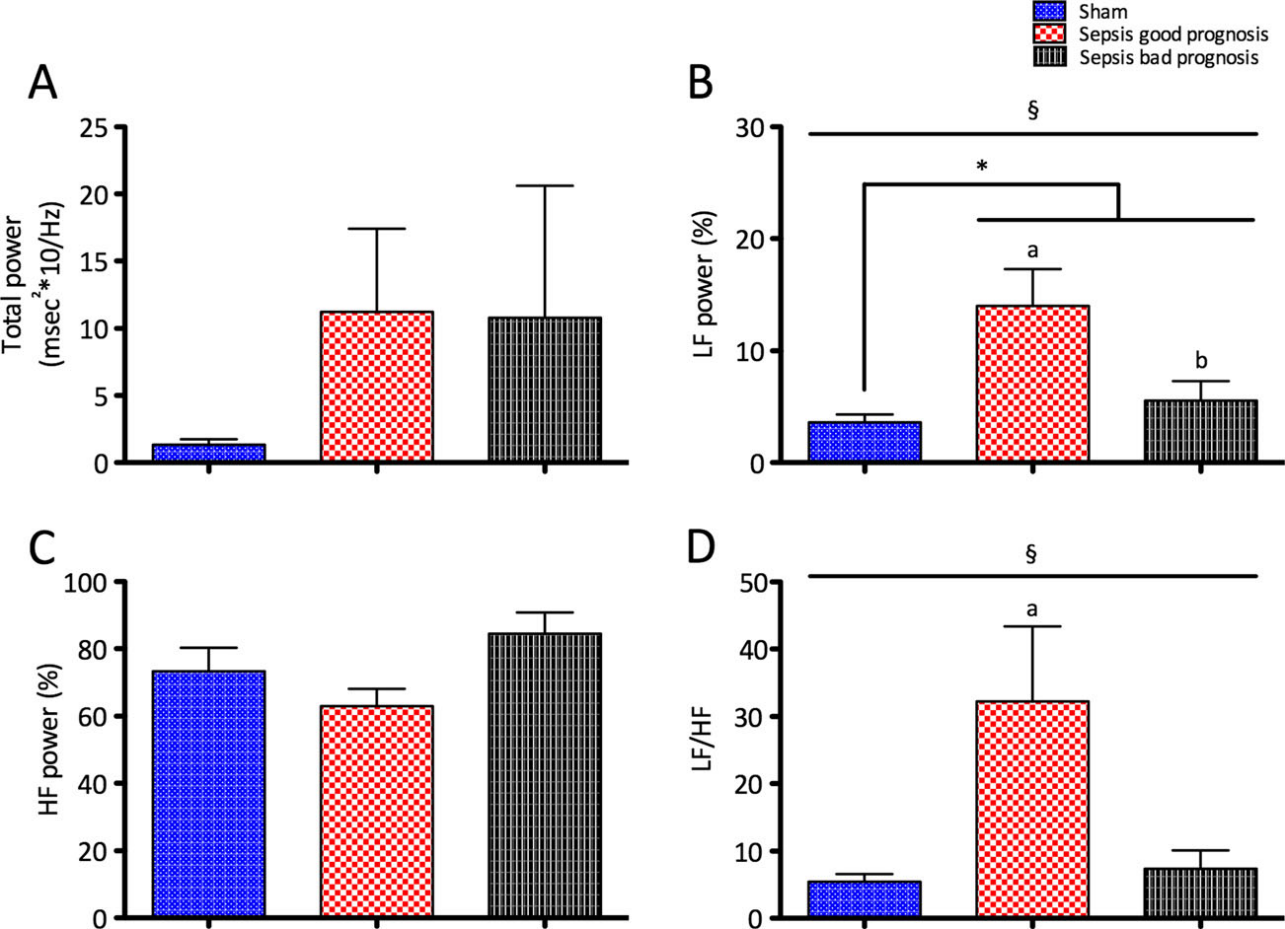
Heart rate variability. **a** Total power is a marker of total autonomic output to the heart. Low-frequency power (LF) (**b**) and LF/HF ratio (**d**) are associated with sympathetic activation, and high frequency power (HF) (**c**) with vagal activation and respiratory activity. Legend: *p < 0.05 for unpaired *t* test (sepsis vs. sham); §*p* < 0.05 for one-way ANOVA; *a p* < 0.05 vs. sham; *b p* < 0.05 vs. sepsis good prognosis. (*n* = 8 for sham, *n* = 6 for sepsis good prognosis, and *n* = 5 sepsis bad prognosis)

We found considerable variation in total power within each experimental group. Differences between groups did not reach statistical significance, despite a tendency to increased variability in sepsis (Fig. 3a). Good prognosis septic animals, a group defined by improved cardiac contractility, had elevated LF power and LF/HF ratio, suggesting increased sympathetic output.

**Fig. 4:**
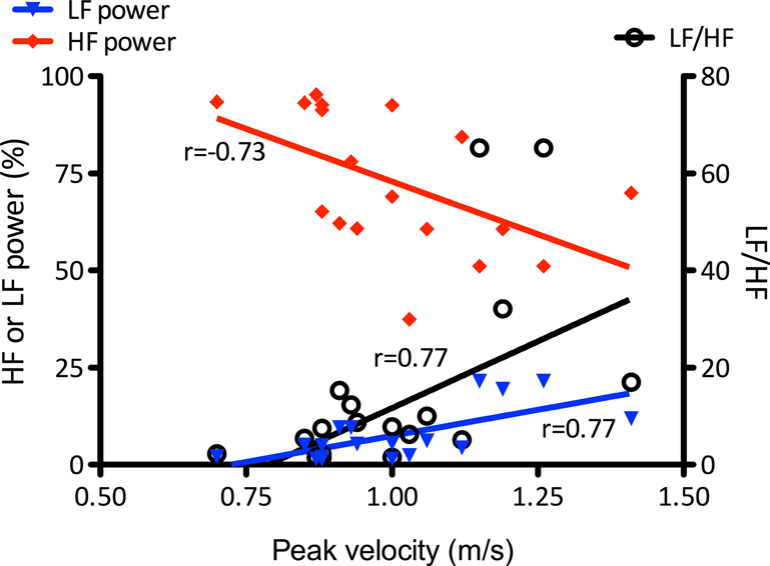
Association between cardiac contractility and indices of autonomic nervous function. Legend: *r*—Spearman correlation coefficient between peak velocity and low frequency power (*LF*, *red diamonds*, *p* = 0.003), high frequency power (*HF*, *blue triangles*, *p* = 0.0011), and LF/HF ratio (*black circles*, *p* = 0.0018) (*n* = 8 for sham, *n* = 6 for sepsis good prognosis, and *n* = 5 sepsis bad prognosis) (color figure online)

Figure 4 illustrates the relationship between aortic peak blood flow velocity (PVel), a marker of left ventricular contractility, and heart rate variability parameters. While LF power and LF/HF positively correlated with PVel (*p* = 0.003 and *p* = 0.0018, respectively), animals with higher HF power, a marker of increased vagal outflow, showed depressed contractility (*p* = 0.0011). There was no significant correlation between the markers of CNS inflammation/injury and cardiac contractility, nor hormone levels; only low levels of correlation were present between the former and the HRV parameters (Supplemental Digital Content).

## Discussion

In this fluid-resuscitated rat model of sepsis, we found evidence of increased inflammation, oxidative injury, and decreased BDNF levels in the hypothalamus and brainstem but not in the pre-frontal cortex, an area not directly involved in autonomic control. Septic animals prognosticated to survive (based on measurements of cardiac contractility at 24 h) had increased sympathetic output. Animals with increased parasympathetic output showed depressed left ventricular contractility. The degree of brain inflammation or injury was not associated (at least not linearly) with either sepsis severity or cardiovascular function.

The relationship between brain inflammation and neuronal damage and the progression of cardiovascular failure in sepsis was originally suggested over 10 years ago in postmortem examinations of different autonomic centers in septic shock patients showing neuronal and glial apoptosis [9]. Increased expression of the inducible form of nitric oxide synthase (iNOS) in the autonomic centers suggested that nitric oxide (NO) released during sepsis could be responsible for impairment of neuronal activation of brain structures related to cardiovascular function [9].

It still remains unclear how brain inflammation directly influences cardiovascular function during sepsis. Brain inflammation, oxidative injury, and BDNF levels are consistently altered in septic animals [5, 24, 25], as well as in the plasma of septic patients that develop brain dysfunction [26, 27]. As these alterations are thought to be involved in the pathophysiology of septic encephalopathy [3], we hypothesized that at least some of these pathways could be involved in the disruption of brain–heart interactions during sepsis. While we did observe changes in these markers within the hypothalamus and brainstem, with decreased BDNF and increased nitrite/nitrate and carbonyl species, we could not demonstrate a specific contribution to changes in HRV and contractility.

Evidence from cecal ligation and puncture (CLP) models suggests early (6 h) activation of neurons in central structures involved in autonomic control and vasopressin release dependent upon NO release and prostaglandin synthesis [28–31]. These findings suggest that brain inflammation is an important event in the modulation of heart function during sepsis. However, these results can be confounded by the presence of early hypotension in these CLP models, particularly with insufficient fluid replacement; early activation of autonomic centers could be secondary to hypotension itself, and not to sepsis [31, 32]. Furthermore, central autonomic activation is reduced in the established phase of sepsis (24 h), an effect likely mediated by NO or leukotrienes [28–31], and associated with a decrease in vasopressin secretion [33, 34]. Inappropriately low levels of vasopressin in septic shock contribute to vascular hyporeactivity [35]. None of these studies used HRV to assess autonomic function, nor did they measure cardiac contractility. The results observed at 24 h post-CLP are consistent with observations in our poor prognosis animals at 24 h; despite the absence of hypotension, there was depressed cardiac contractility and low plasma levels of vasopressin. However, we failed to observe any significant correlation between measures of brain inflammation and either heart rate variability, vasopressin levels, or cardiac contractility.

Diverse effects of inflammation and experimental sepsis on HRV have been reported, likely reflecting the influence of species, type and severity of insult, and the techniques used to measure HRV. Following endotoxemia in rabbits, sympathetic modulation (LF power) of heart rate was impaired with no alterations in plasma catecholamine levels [36]. In endotoxemic rats, loss of vagal tone aggravated systemic inflammation and cardiac impairment with vagal stimulation improving hemodynamics [37]. In a rodent CLP model, there was a reduction in overall HRV, LF power, the LF/HF ratio of HR, and the LF power of MAP, suggesting impaired autonomic control of the heart and circulation which likely contributed to the observed decrease in baroreflex sensitivity [32]. None of these studies analyzed markers of brain inflammation to correlate against alterations in autonomic control. In an overall intensive care population, a low total power of HRV and a high HF/LF ratio (indicating a relative lack of sympathetic tone) were associated with increased mortality while a low HF/LF ratio (relatively high sympathetic tone) was associated with increased survival [12]. Septic shock is associated with a depressed LF power of the heart rate [38]. In septic patients, LF power and the LF/HF ratio were negatively correlated with plasma levels of CRP and IL-10 [39].

In the current study, we demonstrate that LF power is associated with a good prognosis, in the absence of hypotension. Furthermore, LF power is directly associated with cardiac contractility, whereas HF power shows an inverse relationship. Contractility was assessed by aortic peak flow velocity, which was also used to classify animals according to prognosis and disease severity. However, none of these variables were associated with markers of inflammation in the CNS areas responsible for autonomic control. Our systematic approach suggests that, despite the occurrence of brain inflammation, even in the absence of hypotension, the control of autonomic centers and cardiac function is not related to the degree of inflammation, but with the severity of the disease. One possible explanation of this apparent paradox is that control of autonomic response and brain inflammation are epiphenomena that are not mechanistically related. In our model, total power of HRV tended to be higher in septic groups compared to sham controls albeit not reaching statistical significance. We cannot exclude that our methodology, including measuring under a short period of light isoflurane anaesthesia, may be relevant as isoflurane has been reported to decrease autonomic nervous system activity in healthy humans in concentrations equivalent to the ones employed in our study (1MAC) [40, 41]. We used isoflurane to allow for examination (echocardiography and ECG) under minimal stress while critically maintaining spontaneous breathing (fundamental for the evaluation of the high frequency component of the HRV spectrum). All animals were submitted to the same isoflurane concentration to avoid confounding effects on outcome variables. We could not measure HRV in awake animals, and we did not want to deepen anaesthesia as this may potentially compromise cardiorespiratory parameters and thus affect HRV.

There are limitations to our findings. Firstly, our study design does not allow us to determine a mechanistic effect. However, we believe that our systematic approach measuring cytokines related to Th1, Th2, and Th17 cells, as well as markers of nitric oxide production and oxidative stress, plus in vivo cardiac and autonomic function, contributes to the understanding of interactions between these factors, though it does not rule out a role for these cells. Secondly, we analyzed one single timepoint after sepsis when a substantial number of animals had already died. It is likely that responses would be dynamic during the early, established, and recovery phases of sepsis. Inflammation in earlier phases of sepsis may have resulted in neuronal damage in the autonomic centers. However, after 24 h of peritonitis, we could prognosticate surviving animals based on cardiac contractility; this offers an important advantage of our model in differentiating the magnitude of change in survivors and non-survivors at this timepoint. Our model received adequate fluid resuscitation and continuous blood pressure monitoring, thus avoiding the effects of prolonged hypotension on the analyzed parameters, a key difference from other reported studies. We do acknowledge that measurements of inflammation and oxidative injury were performed in the whole hypothalamus and brainstem and not limited to the centers of autonomic control. Finally, the absence of significant correlations between different sets of data suggests the absence of a linear relationship but does not exclude other types of association.

## Conclusion

In our clinically relevant, fluid-resuscitated model of fecal peritonitis, control of autonomic centers and cardiac function was not directly related to the degree of inflammation, but to the severity of sepsis.

## Electronic supplementary material

Below is the link to the electronic supplementary material.

ESM 1 Spearman correlation coefficients between markers of CNS inflammation and injury and peak blood flow velocity, heart rate variability indices and hormone levels (DOC 53 kb)
