# Supplementary material for: Characterization of Brain–Heart Interactions in a Rodent Model of Sepsis
Source: Mol Neurobiol. 2016 May 26;54(5):3745–52. doi: 10.1007/s12035-016-9941-z (PMC5443875; doi:10.1007/s12035-016-9941-z)
Supplement: Supplementary file 1 — Spearman correlation coefficients between markers of CNS inflammation and injury and peak blood flow velocity, heart rate variability indices and hormone levels (DOC 53 kb) [file 12035_2016_9941_MOESM1_ESM.doc]

**Supplemental digital content**

Spearman correlation coefficients between markers of CNS inflammation and injury and peak blood flow velocity, heart rate variability indices and hormone levels. TNF-alpha, IL-1, IL-6, IL-10, BDNF, adrenaline and vasopressin are expressed in pg/ml. Nitrite+nitrate and carbonyl groups are expressed in nmol/mg protein. Total power is expressed in msec*10/Hz and low frequency (LF) and high frequency (HF) power as a percentage of total power (see methods for more details). Peak velocity was expressed in m/s. Legend: * - p<0.05. (n=15).

|  |  | Contractility | Heart rate variability | | | | Hormones | |
| --- | --- | --- | --- | --- | --- | --- | --- | --- |
|  |  | Pvel | Total Power | LF Power | HF Power | LF/HF | Vasopressin | Adrenaline |
| Hypothalamus | TNF-alfa | ns | ns | ns | ns | ns | ns | ns |
|  | IL-1 | ns | ns | ns | ns | ns | ns | ns |
|  | IL-6 | ns | ns | ns | ns | ns | ns | ns |
|  | IL-10 | ns | ns | ns | ns | ns | ns | ns |
|  | BDNF | ns | ns | ns | ns | ns | ns | ns |
|  | Nitrite+Nitrate | ns | ns | **0.554*** | ns | ns | ns | ns |
|  | Carbonyl groups | ns | ns | ns | ns | ns | ns | ns |
| Brainstem | TNF-alfa | ns | ns | ns | ns | ns | ns | ns |
|  | IL-1 | ns | ns | ns | ns | ns | ns | ns |
|  | IL-6 | ns | **0.631*** | ns | ns | ns | ns | ns |
|  | IL-10 | ns | ns | ns | ns | ns | ns | ns |
|  | BDNF | ns | ns | ns | ns | ns | ns | ns |
|  | Nitrite+Nitrate | ns | ns | ns | ns | **0.617*** | ns | ns |
|  | Carbonyl groups | ns | ns | ns | ns | ns | ns | ns |
